# Back pain in elite sports: A cross-sectional study on 1114 athletes

**DOI:** 10.1371/journal.pone.0180130

**Published:** 2017-06-29

**Authors:** Daniela Fett, Katharina Trompeter, Petra Platen

**Affiliations:** Department of Sports Medicine and Sports Nutrition, Ruhr-University Bochum, Bochum, Germany; Oklahoma State University, UNITED STATES

## Abstract

**Objectives:**

To establish the prevalence of back pain in German elite athletes; examine the influence of age, sex, sports discipline and training volume; and compare elite athletes with a physically active control group.

**Methods:**

A standardized and validated online back pain questionnaire was sent by the German Olympic Sports Confederation to approximately 4,000 German national and international elite athletes, and a control group of 253 physically active but non-elite sports students.

**Results:**

We received responses from 1,114 elite athletes (46.5% male and 53.1% female, mean age 20.9 years ± 4.8 years, mean height 176.5 ± 11.5 cm, mean weight 71.0 ± 10.3 kg) and 166 physically active sports students (74.7% male and 24.1 female, mean age 21.2 ± 2.0 years, mean height 180.0 ± 8.0 cm, mean weight 74.0 ± 14.5 kg). In elite athletes, the lifetime prevalence of back pain was 88.5%, the 12-month prevalence was 81.1%, the 3-month prevalence was 68.3% and the point prevalence was 49.0%, compared with 80.7%, 69.9%, 59.0% and 42.8%, respectively in the control group. The lifetime, 12-month and 3-month prevalences in elite athletes were significantly higher than in the control group. Regarding the individual sports disciplines, the prevalence of back pain was significantly higher in elite rowers, dancers, fencers, gymnasts, track and field athletes, figure skaters and marksmen, and those who play underwater rugby, water polo, basketball, hockey and ice hockey compared with the control group. The prevalence of back pain was significantly lower in elite triathletes.

**Conclusions:**

Back pain is a common complaint in German elite athletes. Low back pain seems to be a problem in both elite athletes and physically active controls. A high training volume in elite athletes and a low training volume in physically active individuals might increase prevalence rates. Our findings indicate the necessity for specific prevention programs, especially in high-risk sports. Further research should investigate the optimal dose-effect relationship of sporting activity for the general population to prevent back pain.

## Introduction

Back pain, especially low back pain is a common complaint in the general population. It causes disability, reduces quality of life and impairs ability to work, constituting a great socioeconomic burden on patients and society, resulting in enormous costs for the health care system [1]. Back pain is also common in athletes, but it is not known how its prevalence compares with the general population—particularly among elite athletes.

The relationship between activity level and back pain reportedly follows a U-shaped curve [2–4]. Many studies have shown that too little *and* too much activity is harmful to spinal health [2, 5–10], but the relationship between sports and spinal health has not been adequately clarified. It is well recognized that sports participation generally influences health in a positive way [3], but there is a lack of knowledge about the optimal dose-effect relationship. In this context it is of particular concern whether elite athletes are exposed to a higher risk of developing back pain compared with physically active individuals. We hypothesize that physically active individuals are in the optimal range of the discussed U-shaped curve and thus might have a lower risk of developing back pain. In contrast, elite athletes might be in the end-range position of this curve. They spend a great deal of time training and competing, both of which subject their bodies to a great deal of mechanical strain and a high level of stress on the musculoskeletal system. The amount of strain on the back depends on the duration, intensity and frequency of training, the sports discipline, the level of competition and the training periods during the year [11, 12]. The extent to which this (often daily) strain predisposes elite athletes to back pain is not known. It has been proposed that this physical stress leads to a higher prevalence of back pain in athletes compared with the general population [10, 13, 14].

The prevalence of back pain in athletes has been investigated in several studies, including a systematic review. Trompeter *et al*. [15] summarized back pain prevalence rates in athletes for different time periods and locations at the spine. But due to methodological heterogeneity of summarized studies, a wide range of prevalence has been reported. The prevalence reported in athletes varies widely depending on the sports discipline and study methodology, including descriptions of the exact area of pain and the frequency, duration, intensity or severity of attacks [15]. For example, Lively [16] reported that the lifetime prevalence of low back pain in soccer players was 1%, while Ng *et al*. [17] reported a lifetime prevalence of low back pain of 94% in male rowers. A survey conducted by Cabri *et al*. [18] found a point prevalence of 18% in basketball players, while Ng *et al*. [17] observed a point prevalence of 64% in male rowers.

Trompeter *et al*. [15] found that the various methodologies used in their summarized studies do not allow a comparison among athletes of different disciplines or within a single discipline, or between athletes and the general population. The authors advocated for the use of valid instruments with an internationally accepted definition of back pain. They also found that there are still some disciplines that are uninvestigated. It remains to be clarified whether there are disciplines with particularly strong potential for inducing or preventing the development of back pain.

Most studies focusing on back pain in athletes have examined only one or a few disciplines, and athletes that were not at elite levels. To the best of our knowledge only two studies have examined a large cohort of elite athletes from different sports disciplines. Müller *et al*. [19] reported a mean point prevalence of back pain of 8% in a large cohort of young elite athletes (n = 2,116, mean age 13.3 years). However, the prevalence of back pain in adult athletes was not addressed. Schulz *et al*. [13] investigated the incidence of low back pain in a cohort of older elite athletes (n = 929, mean age 21.4 years) and found a value of 55%. Unfortunately, their study used a self-developed and unvalidated instrument, which contributed to the aforementioned methodological problems [15].

Although many efforts have been made to quantify the prevalence of back pain in elite athletes, it remains unclear whether elite athletes as a group are at increased risk compared with the general population, or non-elite physically active individuals. It also remains unclear whether there are differences among athletes from different sports disciplines. Therefore, we examined the prevalence of back pain in a large cohort of adult professional elite athletes and a control group of non-professional physically active individuals using a valid instrument with an internationally accepted definition for back pain. Additionally, we examined the exact location of pain to get information about which part of the spine is affected in which sport. The size of our cohort was sufficient to allow us to consider athletes from a variety of disciplines, and to identify risk factors.

## Materials and methods

### Study design

A survey of elite athletes competing at national or international level in different sports was conducted. A link to an online questionnaire was sent in January 2015 by e-mail by the German Olympic Sports Confederation using their database of approximately 4,000 elite athletes. The online questionnaire was available until March 2015. The questionnaire was also sent to a group of 253 physically active but non-elite sports students. All participants were informed about the study in a cover letter, and a consent form describing the purposes and procedures of the study was also distributed. Conduct of the study was approved by the regional committee for research ethics and the German Olympic Sports Confederation.

### Back pain questionnaire

The design of the study questionnaire was based on validated, standardized and internationally accepted questionnaires. The instrument was divided into three parts. The first part was based on the standardized Nordic Questionnaire developed and validated to study the prevalence of occupational symptoms [20, 21]. This questionnaire includes several questions about back pain, including separate questions about the neck, upper back and lower back. The term ‘back pain’ was used if the pain occurred in at least one part of the back (neck, upper back, lower back). Questions relating to pain focused on the lifetime (pain at least once in their life), 12-month, 3-month and point prevalence, defined as pain during the last 7 days. Pain was defined as pain, ache or discomfort in an area shown on a diagram of the human body. The second part of the survey consisted of the questionnaire devised by von Korff *et al*. [22] for grading the intensity of chronic pain, and asks respondents to rate the intensity of current pain, the worst and the average pain during the past 3 months on an 11-point numeric rating scale with 0 representing no pain and 10 representing the worst imaginable pain. In addition to the standard questions, questions on symptoms related to sports activity were developed and thoroughly pilot tested (third part of the survey). These include the following:

- What kind of sport are you doing?
- How many years have you been practicing your main sport?
- What is your current level of competition?
- How often and how long do you train during the week?

### Statistical analysis

Statistical analysis was undertaken using SPSS software (version 23, IBM, Armonk, US). Respondents’ characteristics are expressed as the mean and standard deviation. All prevalence data and response rates were rounded to the nearest integer. Prevalence data of athletes from different disciplines were compared when a minimum sample size of n = 15 was reached for any given discipline. Group means were compared using unpaired *t* tests for age, height, weight and training volume, and using Pearson’s chi squared test for sex. Differences in the prevalence of back pain between athletes competing in different sports disciplines (n ≥15) and controls were assessed using the chi-squared test. An unpaired *t* test was used to determine differences in the intensity of back pain between elite athletes and physically active controls, and differences in the duration of back pain were tested with the chi-squared test of goodness of fit. Correlations between back pain, age and training volume were calculated using point-biserial correlation. Odds ratios are reported with 95% confidence intervals (CI). Statistical significance was defined as a p value <0.05.

## Results

Responses from 1,237 elite athletes and 187 physically active controls were received (response rates of 31% and 74%, respectively). Among elite athletes, only squad athletes (A, B, C or D grades) and athletes participating in the 1st or 2nd national divisions who were at least 13 years old were included in the analysis, leading to the exclusion of 123 athletes due to a lower competition level or younger age. We also excluded 21 subjects from the control group who reported being competitive athletes at an elite squad level. The final sample consisted of 1,114 elite athletes and 166 non-elite physically active controls. The characteristics of all respondents are shown in Table 1. Significant between-group differences were observed for mean age, weight and height. The proportion of males in the control group was significantly higher than in the elite group (75% compared with 47%). The training volume was significantly higher in elite athletes.

**Table 1:** Subjects characteristic.

	Athletes (N = 1114)	Controls (N = 166)	P value
Age [years]	20.9 ± 4.8	21.2 ± 2.0	< 0.001
Height [cm]	176.5 ± 11.5	180.0 ± 8.9	< 0.001
Weight [kg]	71.0 ± 14.5	74.0 ± 10.3	< 0.001
Gender (m/f) [%]	46.5/53.1	74.7/24.1	< 0.001
Training volume [h/wk]	18.2 ± 7.7	10.8 ±5.0	< 0.001

f = female, m = male, h/wk = hours per week

### Prevalence of back pain

An overview of the responses to questions concerning back pain is shown in Table 2 for all participating sports disciplines. In elite athletes, lifetime prevalence of back pain was 89%, 12-month prevalence was 81%, 3-month prevalence was 68% and point prevalence was 49%. The lifetime prevalence was significantly lower in the physically active control group (81%, p = 0.005), as were the 12-month (70%, p = 0.001) and 3-month (59%, p = 0.018) prevalences. There was no significant difference in the point prevalence (43% in the control group).

**Table 2:** Overview of responses to questions concerning back pain.

	N	Age (years)	Height (cm)	Weight (kg)	TV (h)	LT-P (%)	12-m P (%)	3-m P (%)	7-d P (%)	worst pain intensity^a^	average pain intensity^a^
**Controls** **All athletes**	**166** **1114**	**21.2** **20.9**	**180.0** **176.5**	**74.0** **71**	**10.8** **18.2**	**80.7** **88.5**	**70.0** **81.1**	**59.0** **68.3**	**42.8** **49.0**	**3.0** **3.8**	**1.8** **2.4**
Archery	7	19.6	178.1	72.1	20.4	85.7	85.7	85.7	85.7	5.1	3.0
Badminton	10	20.4	172	64.3	24.4	80.0	80.0	60.0	70.0	5.5	3.5
Basketball	21	19.2	184.6	77.0	15.5	90.5	90.5	76.2	66.7	4.2	2.5
Beachvolleyball	10	22.3	189.2	77.9	19.2	90.0	80.0	70.0	60.0	4.5	2.7
Bobsleigh	7	24	177.9	82.7	24.0	100.0	100.0	85.7	57.1	3.4	2.0
Boxing	7	23.6	173.6	70.8	21.8	71.4	57.1	57.1	57.1	3.3	2.6
Canoe	33	22.5	177.6	73.8	19.8	93.9	84.8	66.7	54.5	4.0	2.8
Curling	12	26.4	177.6	80.2	14.4	91.7	91.7	58.3	41.7	3.7	2.4
Cycling	29	20.1	176.1	68.4	18.9	86.2	82.8	72.4	55.2	3.4	2.2
Dancing	22	22.5	176.7	66.2	15.7	95.5	90.9	77.3	59.1	4.5	2.5
Diving	10	20.3	174.4	70.5	25.5	100.0	80.0	70.0	40.0	2.8	1.8
Fencing	23	22.1	177.3	70.0	20.2	100.0	95.7	78.3	34.8	4.4	3.0
Figure skating	15	18.7	168.4	60.3	20.2	93.3	80.0	86.7	66.7	5.0	3.4
Golf	1	31.0	160.0	58.0	4.0	100.0	100.0	100.0	0.0	3.0	1.0
Gymnastics	32	17.3	165.6	55.6	23.7	93.8	87.5	68.8	46.9	5.2	3.3
Handball	31	18.5	177.2	72.0	13.9	83.9	83.9	64.2	35.5	4.4	2.9
Hockey	116	19.9	173.1	66.0	15.0	86.2	82.8	66.4	44.8	3.3	2.1
Horseriding	8	20.4	171.3	59.0	19.4	87.5	87.5	87.5	75.0	4.9	3.3
Ice hockey	27	21.4	172.0	68.6	19.1	88.9	85.2	81.5	63.0	4.7	3.4
Judo	34	19.9	171.0	69.5	19.2	91.2	79.4	73.5	55.9	3.8	2.5
Karate	28	18.3	167.4	58.8	11.8	78.6	71.4	50.0	35.7	2.8	2.0
Luge	9	20.2	179.2	77.8	21.5	100.0	100.0	100.0	66.7	4.6	2.9
Modernern pentathlon	2	19.5	173.0	62.0	21.5	100.0	100.0	100.0	100.0	7.0	7.0
Rowing	83	21.1	183.3	76.6	19.6	96.4	95.2	79.5	67.5	4.6	2.7
Rugby	30	22.2	179.2	82.3	15.8	83.3	73.3	50.0	30.0	3.1	1.8
Sailing	6	23.5	169.3	66.7	23.8	83.3	83.3	50.0	0.0	3.0	0.8
Shooting	23	23.3	175.4	71.2	17.7	95.7	87.0	73.9	69.6	4.6	2.8
Skiing	49	19.8	175.6	67.5	21.9	87.8	73.5	65.3	44.9	3.4	2.0
Soccer	2	21.5	163.0	55.5	16.3	100.0	100.0	100.0	50.0	3.5	1.5
Speed skating	33	20.1	176.4	70.5	25.5	93.9	84.4	75.8	51.5	3.7	2.3
Swimming	45	19.7	178.8	70.4	20.0	88.9	73.3	68.9	37.8	3.8	2.5
Synchronised swimming	3	20	173.3	57.3	17.3	66.7	66.7	33.3	33.3	3.5	1.5
Tabletennis	1	20.0	188.0	84.0	40.0	0.0	0.0	0.0	0.0	0.0	0.0
Taekwondo	10	21.3	173.3	63.3	25.7	90.0	90.0	90.0	70.0	4.6	3.6
Tennis	14	16.1	175.4	66.4	19.9	85.7	78.6	71.4	42.9	3.9	2.6
Track and field	99	20.5	178.7	72.5	17.7	86.9	83.8	63.6	44.4	3.5	2.3
Triathlon	16	17.3	173.1	57.7	18.7	56.3	43.8	37.5	31.3	1.9	1.3
Underwater rugby	29	25.7	179.6	76.0	11.6	89.7	89.7	79.3	58.6	4.3	2.6
Volleyball	36	20.7	191.2	81.9	22.1	91.7	69.4	50.0	27.8	2.6	1.6
Waterpolo	19	23.4	179.7	76.9	20.3	100.0	89.5	84.2	73.7	5.5	3.8
Weightlifting	35	23.8	172.7	83.6	17.1	82.9	71.4	60.0	42.9	3.5	2.3
Wrestling	18	20.3	171.5	70.1	15.2	77.8	66.7	66.7	50.0	3.2	2.2

a = concerning the last 3 month

### Intensity of back pain

Results concerning the intensity of back pain are shown in Table 2. On an 11-point numeric rating scale, the worst intensity of back pain during the last 3 months was 3.8 in athletes and 3.0 in active controls (p < 0.001). Values of the average pain intensity were 2.4 and 1.8 (p = 0.001), respectively.

### Location and duration of pain

An overview of the location of pain is shown in Table 3. The distribution of back pain location was identical for elite athletes in each discipline. The low back was the most commonly affected area for all time periods in elite athletes and physically active controls (lifetime prevalence 77% and 71%, 12-month prevalence 65% and 59%, 3-month prevalence 50% and 46%, and point prevalence 34% and 29%, respectively).

**Table 3:** Location of back pain.

	Neck			Upper back			Lower back		
	*Athletes*	*Controls*	*p*	*Athletes*	*Controls*	*p*	*Athletes*	*Controls*	*p*
Lifetime [%]	63	50	0.001	46	39	n.s.	77	71	0.045
12-month [%]	52	39	0.001	36	27	0.009	65	58	ns
3-month [%]	37	30	0.035	27	22	n.s.	50	46	ns
7-day [%]	23	22	n.s.	16	15	n.s.	34	29	ns

ns = not significant

The next commonly affected area was the neck (lifetime prevalence 63% and 50%; 12-month prevalence 52% and 39%; 3-month prevalence 37% and 30%; and point prevalence: 23% and 22%, respectively).

The lowest prevalence was found for the upper back (lifetime prevalence 46% and 39%; 12-month prevalence 36% and 27%; 3-month prevalence 27% and 22%; and point prevalence 16% and 15%, respectively).

Results concerning the duration of pain are presented in Table 4. There are significant differences in the distribution of the pain duration.

**Table 4:** Duration of pain.

		3-m prevalence [%]		12-m prevalence [%]		
	*Athletes*	*Controls*	*p*	*Athletes*	*Controls*	*p*
*Duration of LBP*:						
1–7 days	31	31	ns	21	21	ns
8–30 days	15	10	< 0.001	26	28	ns
> 30 days, but not daily	5	2	< 0.001	18	9	< 0.001
daily	1	2	0.023	2	3	0.020
*Duration of NP*						
1–7 days	29	27	ns	26	25	ns
8–30 days	8	6	< 0.001	18	13	0.005
> 30 days, but not daily	3	2	ns	10	8	0.015
daily	2	2	ns	2	2	ns
*Duration of TP*						
1–7 days	18	13	< 0.001	18	12	< 0.001
8–30 days	7	4	< 0.001	12	10	ns
> 30 days, but not daily	2	1	0.036	6	4	< 0.001
daily	1	1	ns	1*	1*	0.039

LBP = low back pain; m = month; NP = neck pain; ns = not significant TP = thoracic pain;

*optical equality because of rounding to the nearest integer. Unrounded values: Athletes: 1.1%; Controls: 0.6%.

### Back pain and age

**Fig 1:**
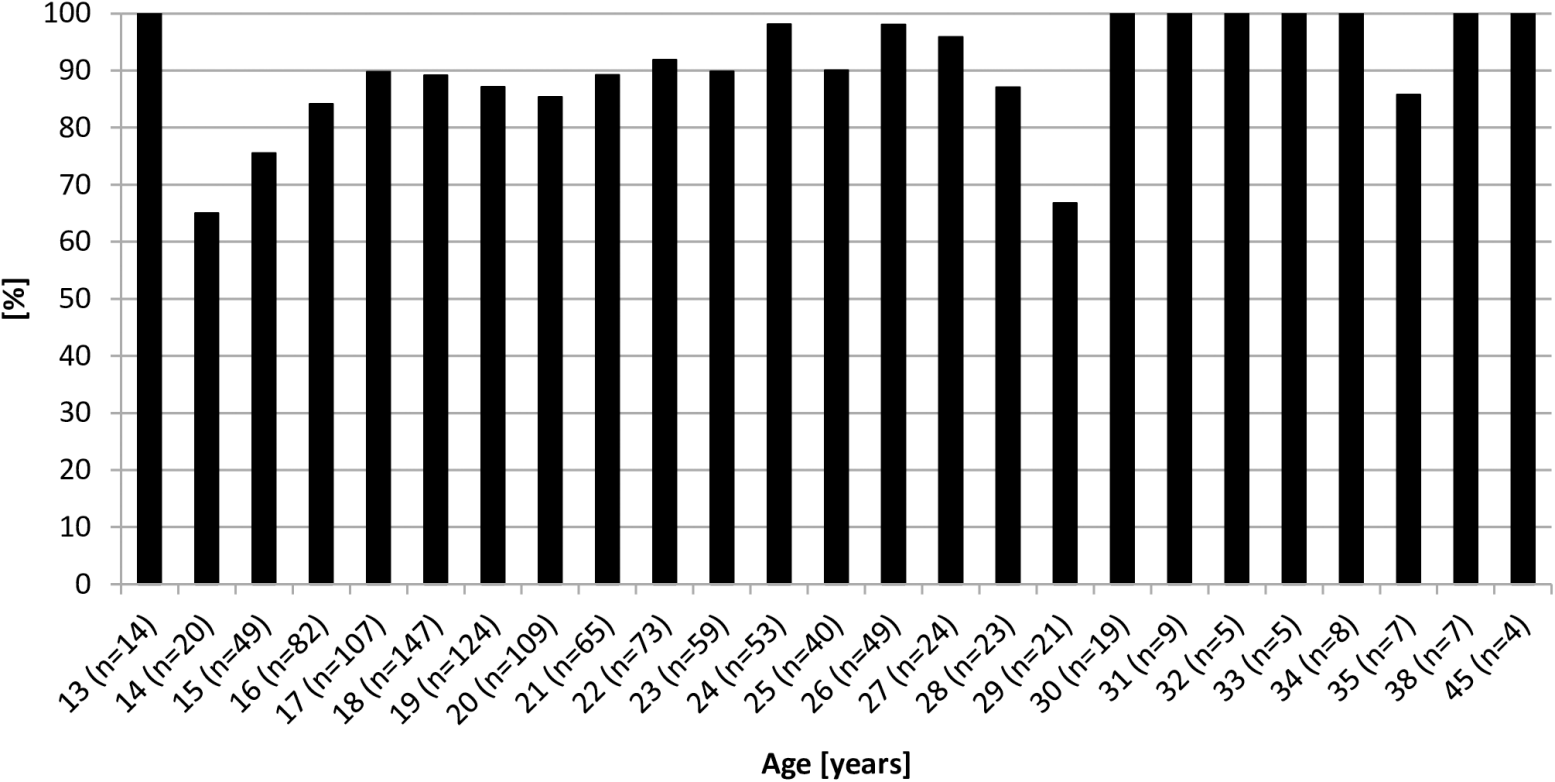
Lifetime prevalence of back pain in athletes categorized by age.

There was a correlation between an elite athlete’s age and the lifetime prevalence of back pain (p <0.001, Fig 1). Lifetime prevalence was 86% in elite athletes aged 13–18 years, increasing to 87% in 19–24 year olds, 89% in 25–30 year olds, and 98% in those older than 30 years. There was no significant relationship between back pain and age in physically active controls.

### Back pain and sex

Female elite athletes had a significantly higher prevalence of back pain than males for the 3-month period (female 71% *versus* male 65%) and 7-day period (females 53% *versus* males 44%). A similar relationship was observed in the physically active control group, with significantly higher prevalence for females for the 12-month, 3-month and 7-day periods (12-months, females 83% *versus* males 66%; 3-months, females 75% *versus* males 54%; point prevalence, females 60% *versus* males 38%).

### Back pain and training volume

The training volume of elite athletes was 18.2 ± 7.7 hours per week and for the control group 10.8 ± 5.0 hours per week. For elite athletes there was a significant positive correlation between back pain prevalence and weekly training volume for the lifetime, 12-month and 3-month time periods (p < 0.05). No correlations were found for the active control group.

### Back pain and sports disciplines

**Fig 2:**
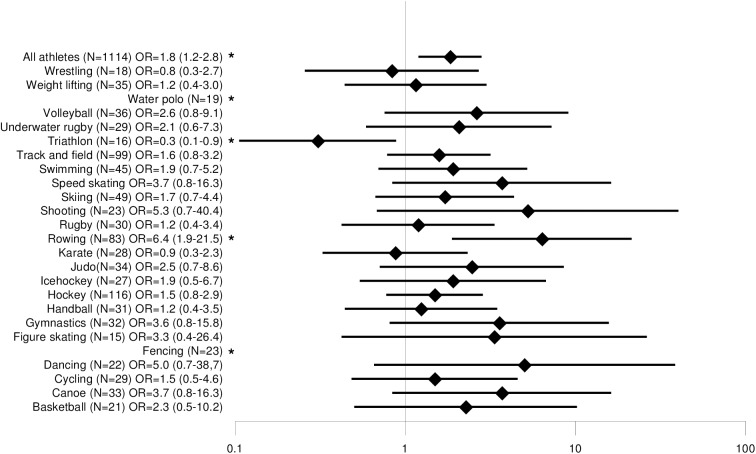
Comparison of lifetime prevalence of back pain odds ratios among different sports.

**Fig 3:**
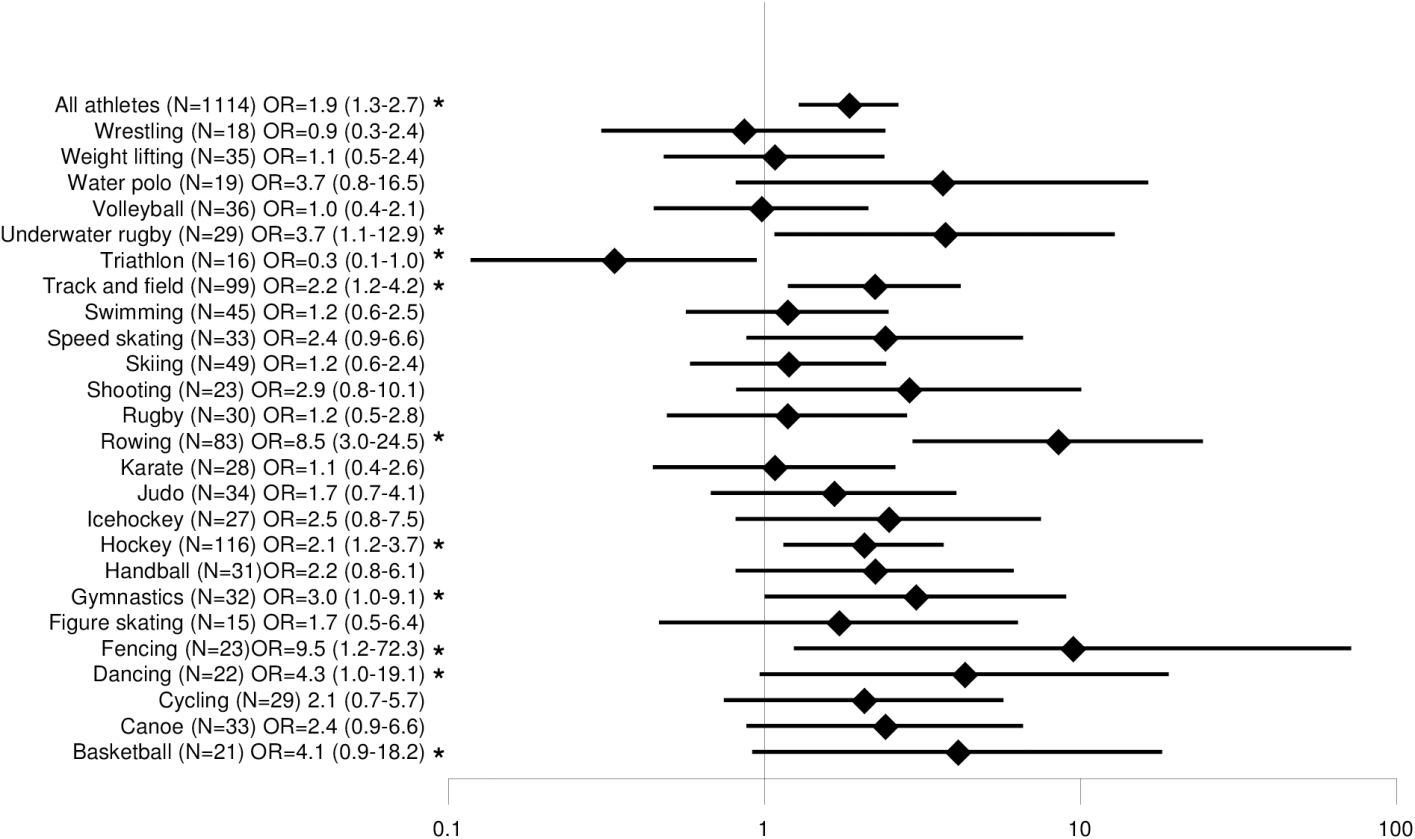
Comparison of 12-month prevalence of back pain odds ratios among different sports.

**Fig 4:**
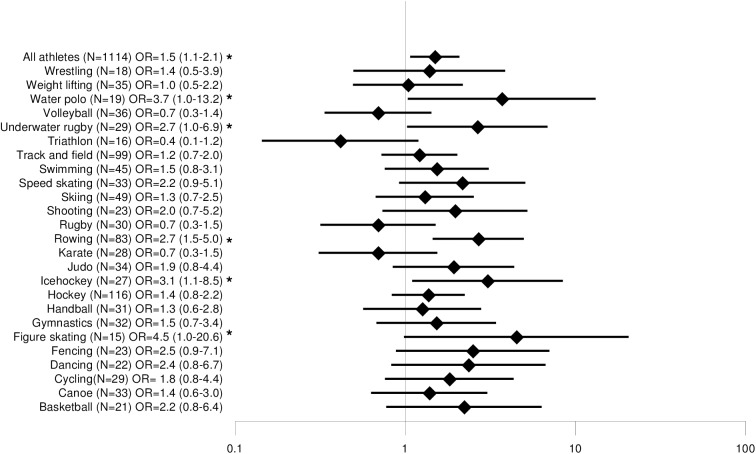
Comparison of 3-month prevalence of back pain odds ratios among different sports.

**Fig 5:**
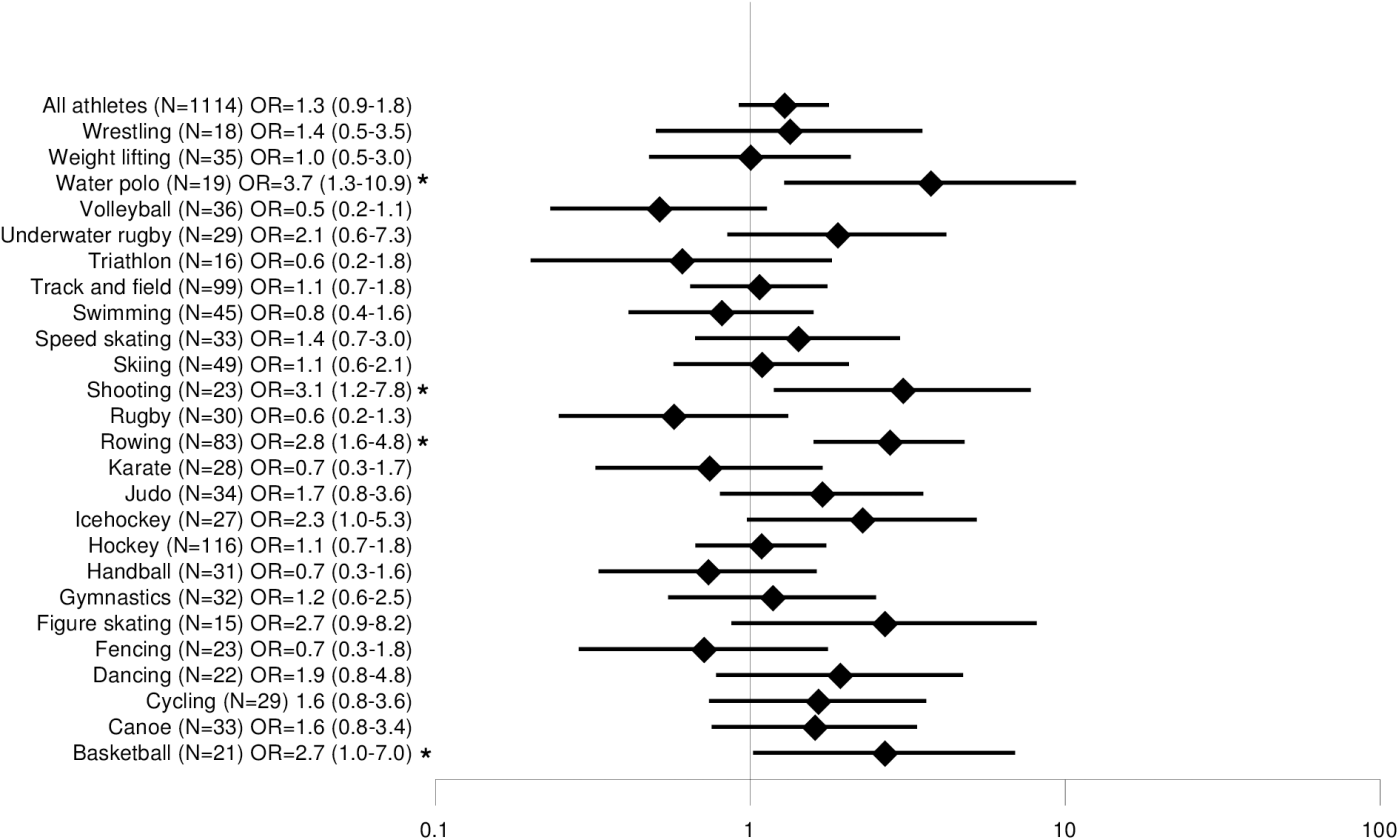
Comparison of 7-day prevalence of back pain odds ratios among different sports.

Disciplines with a minimum sample size of n = 15 were compared with each other. Lifetime prevalence of back pain ranged from 56% (triathlon) to 100% (diving, fencing, water polo), 12-month prevalence from 44% (triathlon) to 96% (fencing), 3-month prevalence from 38% (triathlon) to 90% (taekwondo) and point prevalence from 28% (volleyball) to 74% (water polo). The odds ratios for back pain are shown in Figs 2, 3, 4 and 5. The odds ratio for back pain among elite triathletes was lower than in physically active controls. The odds ratios for back pain were significantly higher in elite athletes who participated in rowing, dancing, fencing, gymnastics, underwater rugby, water polo, shooting, basketball, hockey, track and field athletics, ice hockey and figure skating in some time periods.

## Discussion

The purpose of this investigation was to evaluate the prevalence of back pain in German elite athletes compared with a physically active control group, and to examine the influence of age, sex, sports discipline and training volume.

Our main findings were: (a) a higher prevalence of back pain among elite athletes compared with physically active controls; (b) the lower back as the main location of back pain in elite athletes of all disciplines and in physically active controls; (c) an increase in back pain prevalence with age in elite athletes; (d) a higher 3-month and point prevalence rate in female elite athletes compared with male elite athletes; and (e) sports-specific differences in the prevalence of back pain.

It was hypothesized that elite athletes would have a higher prevalence of back pain compared with a physically active control group. Indeed, the prevalence of back pain was significantly higher in the group of elite athletes. The degree of stress on the musculoskeletal system during sporting activities on such a highly competitive level might explain the high prevalence rates. Physically active individuals had a significantly lower weekly training volume and thus a lower level of stress on the musculoskeletal system due to sports activities. The findings underline the hypothesis that the controls were closer to optimal levels of activity compared with the elite athletes. It remains unclear how various recreational sports should be ranked on their risk factors for back pain. According to the U-shaped curve of Heneweer *et al*. [3], we think that different sports disciplines can influence health positively and assist in preventing back pain, if performed moderately.

### Location

The main location of back pain was the lower back for elite athletes of all disciplines and for physically active controls. In the literature, low back pain also seems to be the most frequent physical complaint for athletes and the general population [13, 18, 23–27]. The thoracolumbar spine is particularly predisposed to injury due to biomechanical factors related to the physiological curves of the spine. In this area the transition from the natural lordosis to kyphosis places special demands on the spine. Forces of axial compression, distraction and rotation affect the spine especially in this area. Additionally, reduced activity of lumbopelvic stabilizing muscles and the high frequency of end-range lumbar spine positions in different sports are associated with a potential risk for lumbar spine injury and low back pain [28–33].

In our investigation there was a significant difference between elite athletes and controls in lifetime prevalence of low back pain. However, other specific time periods showed no significant differences; low back pain was a big problem in both groups. This indicates that there might also be risk factors for the control group to develop back pain in this area. However, our results concerning duration and intensity showed differences between the groups.

The prevalence of back and low back pain that we observed in our entire cohort of elite athletes appears to be higher than that in the general population [23, 24]. Walker [23] summarized 27 studies and reported a lifetime prevalence of low back pain in the general population ranging from 11% to 84%. Only two studies showed a higher prevalence (79% and 84%) than our findings for athletes (77%), while the other 25 studies showed lower values. Additionally, in another population-based review, Hoy *et al*. [24] calculated a mean prevalence of 39% ± 24%, which is also much lower than our results.

With respect to 12-month prevalence in the general population, Walker [23] summarized 16 studies and showed a range of 10% to 65%. Two of these studies had values that are as high as our findings for athletes; the other 14 studies found lower prevalence values.

The point prevalence of low back pain in the general population reportedly ranges from 7% to 33% [23], with a mean point prevalence of 18% ± 12% [24]. Our investigation revealed a higher point prevalence of low back pain for athletes than has been observed in any of these population-based studies.

Although the prevalence of back and low back pain that we observed in elite athletes appears to be higher than that in the general population, such comparisons must be interpreted with caution, due to methodological differences in the studies.

### Back pain and age

A frequently discussed confounder for back pain is age. In the general population, the prevalence of back pain in children and adolescents is reportedly lower than that seen in adults. It rises with age and peaks at 55–64 years [10, 34]. We also found a relationship between age and back pain in elite athletes, findings that chime with those of Müller *et al*. [19], but we did not observe this relationship in the physically active control group. This finding could be explained by the relatively small age range of our control group respondents.

### Back pain and sex

In the general population, back pain is reported more commonly in females than in males. A frequently discussed explanation for this phenomenon is the earlier maturity of girls, or their hormonal changes during puberty compared with boys [35]. Also the anatomical characteristics of the female body have been implicated as reinforcing the development of back pain and therefore leading to higher prevalence in females than males [35]. In this context, several studies have discussed menstrual low back pain, pregnancy-related back pain, or the lower muscle mass and bone densities of females that might result in destabilization of the body and thus insufficient compensation for high loads [36, 37]. There may also be social and educational explanations; it may be more socially acceptable for women to report their symptoms than men. Finally, as Shan *et al*. [38] reported, boys might have a higher pain threshold than girls.

However in athletes, the relationship between back pain and sex or gender is controversial. Some studies have reported that adolescent and adult female athletes are more likely to report back pain [9, 35, 38–42], while others have found higher rates for males than for females [17, 43–45]. Sex differences in the prevalence of musculoskeletal pain in elite athletes might be influenced by different factors. In some disciplines, male athletes might tolerate higher loads because of their higher training volume or higher loads during strength training, or because of differences in basic rules (e.g., the number of sets in tennis). Additionally, differences in spinal kinematics have been reported for some disciplines, and a link between spinal kinematics and back pain has been suggested [46]. However, in our investigation, female elite athletes reported a higher prevalence of back pain during the last 3 months and during the last 7 days than did male elite athletes. No differences for lifetime or 12-month prevalence were found.

### Risk factors for back pain in sports

In general, studies focusing on back pain in sports have suggested that factors such as high training volume, repetitive motions, high physical loads, repetitive mechanical strain and extreme body positions might be responsible for high prevalence of back pain [47–49].

Regarding the training volume in this investigation, there was a significant correlation between back pain prevalence and weekly training hours in the group of elite athletes. In the physically active control group, such a correlation was not found; both high and low amounts of sporting activity appeared to predispose respondents to back pain in this group. Control group respondents with a weekly training volume <3 hours had a lifetime prevalence that was similar to that of elite athletes with high training volumes. These findings agree with those of Heneweer *et al*. [3], who identified a U-shaped relationship between athletic activity and back pain. As the prevalence of back pain varied enormously between different disciplines with similar training volumes, we judge that the intensity and the content of training, and the physical and psychological constitution of an athlete, are likely also to be highly influential. These will be important areas of further study.

Generally, with regard to prevalence and training volume of athletes, it must be recognized that both vary during the season [12]. Newlands *et al*. [12] found a high variability (6%-25%) in monthly LBP prevalence during a 12-month period among international-level rowers. The highest rates were observed during the peak season. These variable rates might be linked to variable training volume. Athletes in our investigation were surveyed between January and March. Further studies should consider the time of examination. Additionally, it would be beneficial to investigate varying volume, intensity and content of training during the season and linking these variables with back pain prevalence to clarify whether there is a direct relationship.

In some disciplines, higher back pain prevalence was found for elite athletes compared with the active control group. The risk factors that have been discussed in the literature might have influenced the prevalence rates in these sports.

For example, rowers, hockey and ice hockey players often train and compete in a hyperflexed position of the trunk. Additionally, they are exposed to high loads due to contact from opponents. In basketball these issues, along with the high frequency of jumps and landings, are also problems that might promote back pain. Dancers, gymnasts and figure skaters may not be able to tolerate the high loads due to extreme body positions, landings after jumps and the high frequency of end-range lumbar spine positions [46]. Other sports disciplines in this investigation show the same risk factors but back pain prevalence does not significantly differ between elites and controls. It may be that the preventive factors in these other sports outweigh the risk factors for back pain. However, it also must be acknowledged that the sample size of nearly all other disciplines in this investigation was very small; therefore, low power may have affected statistical significance.

### Sport-specific prevalence of back pain

There was wide variability of prevalence rates reported by athletes of different disciplines. Our results must be interpreted with caution, however, as in some disciplines there were very small sample sizes, increasing our confidence intervals in the odds ratios. For some sports disciplines, however, nearly all of the squad athletes participated, so the sample size was close to the size of the total population of German elite squad members in these disciplines.

We found significantly higher rates of back pain in those who participate in elite rowing, dancing, fencing, gymnastics, underwater rugby, water polo, shooting, basketball, hockey, track and field athletics, ice hockey and figure skating; only elite triathletes exhibited a significantly lower prevalence compared with controls. The previously reported prevalence of back pain in athletes of specific disciplines has varied widely, likely due to the methodological heterogeneity of studies. Nevertheless, the low prevalence of back pain we observed in elite triathletes reflects the findings of other investigators. Villavicencio *et al*. [50, 51] reported a lifetime incidence for low back pain of 69% in triathletes, while Manninen and Kallinen [52] reported a lifetime incidence of 59%, a 12-month incidence of 32% and a point incidence of 12%. It has been proposed that running and swimming is not the primary cause for back pain in triathletes; Triki *et al*. [40] have suggested that swimming might prevent back pain while cycling may provoke it [52]. It is possible that the variation in training disciplines in triathlon has a positive influence on the development of back pain, but this hypothesis will require further research. Consistent with findings in the literature [12, 17, 47, 53], our investigation revealed that rowing is associated with a very high prevalence of back pain, suggesting that high training volume, high loads during resistance training and highly repetitive movements might be responsible. In this context, Howell [47] reported a strong relationship between excessive lumbar flexion and the incidence of low back pain or discomfort in a group of elite lightweight female rowers, and suggested that mechanical stress on non-contractile tissue sufficient to stimulate musculoskeletal pain receptors in the low back could result from intermittent or continuous hyperflexion of the lumbar spine.

### Limitations

Our findings may have been influenced by recall bias, which is a particular concern in any retrospective cross-sectional study. Our questionnaire did not illuminate how pain developed or its associated factors; longitudinal studies of these variables would be of great interest [42, 54]. There may also have been a response bias caused by acquiescence, socially desirable responding or extreme responding. Both causes of biases could have caused us to over- or underestimate the prevalence of back pain. Athletes with back pain may have been more likely respond to our survey, so our findings should be interpreted with caution. The response rate of athletes in our survey was in line with previous studies in German elite athletes [13], but was low compared with many international studies on back pain in athletes on different competition levels [12, 21, 25–27, 43, 45, 55–57]. Additionally, many studies reporting on prevalence of back pain in athletes do not mention response rates [16, 47, 53, 58–66]. However, the difference in response rates between our two groups is very large, and this may have influenced the results.

Also, the analysis of prevalence rates in different sports disciplines should be interpreted carefully as it may have been affected by sample size effects. The comparison between elite athletes and physically active controls must be interpreted in the context of the significant between-group differences in age, anthropometrics and sex. Further studies should examine age- and sex-matched control groups, ideally also with comparable anthropometric characteristics.

## Conclusions

Back pain is a common complaint in German elite athletes. Low back pain seems to be a problem in both elite athletes and physically active controls. Prevalence data gave a first indication that both a very active and a sedentary lifestyle increase the prevalence of back pain. The high training volume of elite athletes might increase prevalence rates, as might the low training volume in physically active or inactive individuals. Further research should investigate the optimal dose-effect relationship of sporting activity for the general population. This would offer the opportunity to enhance health in general and to prevent back pain. Our findings indicate the necessity for specific back pain prevention programs, especially in high-risk sports. Athletes, physicians, physiotherapists and coaches should be aware of this, and seek to identify specific prevention programs. Back pain intervention programs should be part of elite athletes’ daily training.

## Supporting information

S1 File Data availability. (SAV)

S2 File 1st part of the questionnaire–English version. (PDF)

S3 File 1st part of the questionnaire–German version. (PDF)

S4 File 2nd part of the questionnaire–German and English version. (PDF)

S5 File 3rd part of the questionnaire–German and English version. (DOCX)
