# Supplementary material for: Back pain in elite sports: A cross-sectional study on 1114 athletes
Source: PLoS One. 2017 Jun 29;12(6):e0180130. doi: 10.1371/journal.pone.0180130 (PMC5491135; doi:10.1371/journal.pone.0180130)
Supplement: S2 File — (PDF) [file pone.0180130.s002.pdf]

Applied Ergonomics 1987, **18.3**, 233–237

Standardised Nordic questionnaires for the analysis of musculoskeletal symptoms

I. Kuorinka*, B. Jonsson†, A. Kilbom**, H. Vinterberg††, F. Biering-Sørensen§, G. Andersson§§ and K. Jørgensen[π]

*Institute of Occupational Health, Department of Physiology, Helsinki, Finland;

†National Board of Occupational Safety and Health, Work Physiology Unit, Umeå, Sweden;

**National Board of Occupational Safety and Health, Work Physiology Unit, Research Department, Solna, Sweden;

††Department of Rheumatology, County Hospital, Hillerød, Denmark;

§Rigshospitalet, University of Copenhagen, Denmark;

§§Department of Orthopaedic Surgery, Rush-Presbyterian – St. Luke's Medical Center, Chicago, USA;

[π]August Krogh Institute, University of Copenhagen, Denmark

Standardised questionnaires for the analysis of musculoskeletal symptoms in an ergonomic or occupational health context are presented. The questions are forced choice variants and may be either self-administered or used in interviews. They concentrate on symptoms most often encountered in an occupational setting. The reliability of the questionnaires has been shown to be acceptable. Specific characteristics of work strain are reflected in the frequency of responses to the questionnaires.

Keywords: Questionnaires, musculoskeletal disorders, occupational health

Background

Musculoskeletal disorders and symptoms in a working population are common, occurring predominantly in the low back (see review by Troup and Edwards, 1985), neck and upper limbs (e g, Armstrong *et al,* 1982; Waris, 1979; Oxenburgh *et al,* 1985). Mechanical factors contribute to the development of these problems and in general influence symptoms (Kilbom *et al,* 1986; Maeda *et al,* 1979; Pope *et al,* 1984). To help define the problem and its relationship to work factors, increasing interest has been directed in many countries to the development of methods to estimate and record musculoskeletal symptoms. Questionnaires have proved to be the most obvious means of collecting the necessary data.

Standardisation is needed in the analysis and recording of the musculoskeletal symptoms. Otherwise it is difficult to compare the results from different studies. This consideration was the main motive for a Nordic group to start developing standardised questionnaires for the analysis of musculoskeletal symptoms. Even a modest degree of standardisation was regarded as useful. We found that the major part of most questionnaires used in previous studies could have been easily comparable, but that the individual questions often differed in trivial details from study to study and thus impeded the comparison of the results. It was evident that the knowledge about the musculoskeletal symptoms was not sufficient to allow an advanced degree of standardisation. Consequently, we faced a trade-off between the banality of the questionnaire and the depth of the approach. The questionnaires presented here are a compromise between the extremes. We are well aware, however, that use of identical questionnaires is not the only prerequisite for comparison of data from different studies.

The questionnaires follow the tradition of some earlier medical questionnaires – e g, for cardiovascular (Rose and Blackburn, 1968) or pulmonary surveys (British Medical Research Council's questionnaire for chronic bronchitis (Anon, 1960a, 1960b)). The nature of the musculoskeletal symptoms dictates a different structure, however.

Supported by the Nordic Council of Ministers, a project was undertaken to develop and test standardised questionnaires on general, low back and neck/shoulder complaints. The text has been translated into four Nordic languages, using a multiple to-and-from technique from the source languages which were Swedish and Danish. Translation into English has been guided by native speakers of English, but might require further revision. If comparability with the Nordic languages is desired, a further check-and-cross translation is recommended.

Structure of the questionnaires

The questionnaires consist of structured, forced, binary or multiple choice variants and can be used as self-administered questionnaires or in interviews. There are two types of questionnaires: a general questionnaire, and specific ones focusing on the low back and neck/shoulders. The purpose of the general questionnaire is simple surveying, while the specific ones permit a somewhat more profound analysis.

The two main purposes of the questionnaires are to serve as instruments (1) in the screening of musculoskeletal disorders in an ergonomics context, and (2) for occupational health care service. The questionnaires may provide means to measure the outcome of epidemiological studies on musculoskeletal disorders. The questionnaires are not meant to provide a basis for clinical diagnosis. Screening of the musculoskeletal disorders may serve as a diagnostic tool for analysing the work environment, workstation and tool design. The incompatibility of the user and the task or the tool have been shown to relate to the musculoskeletal symptoms (van Wely, 1970; Corlett and Bishop, 1978). The localisation of symptoms may reveal the cause of loading. The occupational health service may use the questionnaire for multiple purposes — e g, for diagnosis of the work strain, for follow-up of the effects of improvements of the work environment, and so on.

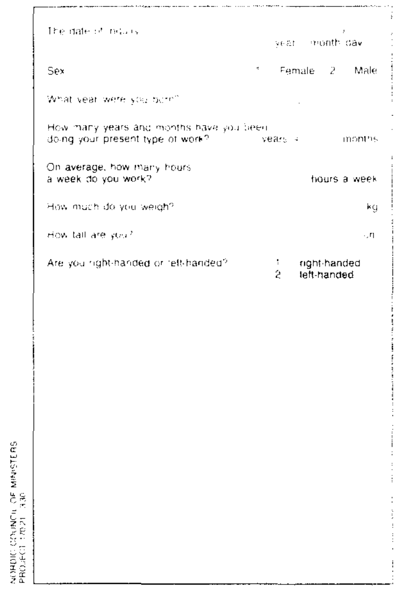

Questionnaire about trouble with the locomotive organs

The date of return — year / month / day

Sex — 1 Female 2 Male

What year were you born?

How many years and months have you been doing your present type of work? — years + months

On average, how many hours a week do you work? — hours a week

How much do you weigh? — kg

How tall are you? — cm

Are you right-handed or left-handed? — 1 right-handed 2 left-handed

NORDIC COUNCIL OF MINISTERS PROJECT 17021 330

Fig. 1 Anatomical areas and questionnaire layout

General questionnaire

The general questionnaire was designed to answer the following question: "Do musculoskeletal troubles occur in a given population, and if so, in what parts of the body are they localised?" With this consideration in mind, a questionnaire was constructed in which the human body (viewed from the back) is divided into nine anatomical regions. These regions were selected on the basis of two criteria: regions where symptoms tend to accumulate, and regions which are distinguishable from each other both by the respondent and a health surveyor. The intentional choice of the back aspect of the body leaves gaps when disorders are located in the frontal part of the shoulder or on the flexor side of the upper limbs. This choice has been made because numerous possible causes of pain in the front part of the body (abdominal and thoracical pains, etc) might intermingle with the musculoskeletal pain in the upper thorax. Preliminary observations seem to point out that this choice does not significantly modify the response rates. The verbal questions deal with each anatomical area in turn, and inquire whether the respondent has, or has had, troubles in the respective area during the preceding 12 months, whether this pain is disabling and whether it is ongoing. Fig. 1 shows the anatomical areas and the layout of the questionnaire.

Special questionnaires for low back, neck and shoulder symptoms

The two specific questionnaires (Figs. 2 and 3) concentrate on anatomical areas in which the musculoskeletal symptoms are most common. These questionnaires probe more deeply into the analysis of the respective symptoms and contain questions on the duration of the symptoms over past time — i e, entire life, last 12 months, and previous 7 days. The main broadening in these questionnaires is that they analyse more thoroughly the severity of the symptoms in terms of their effect on activities at work and during leisure time, and in terms of total duration of symptoms and sick-leave during the preceding 12 months.

Limitations of the questionnaires

The general limitations of questionnaire techniques also apply to these standardised questionnaires. The experience of the person who fills out the questionnaire may affect the results. Recent and more serious musculoskeletal disorders are prone to be remembered better than older and less serious ones. The environment and filling out situation at the time of the questioning may also affect the results (Brigham, 1975; Sinclair, 1975). From an epidemiological viewpoint, it is evident that this type of questionnaire is most applicable for cross-sectional studies with all the concomitant limitations.

Experience from the use of the questionnaires

The standardised questionnaires have been in extensive use in Denmark, Finland, Norway and Sweden. The questionnaires, mainly the general questionnaire, have been used in more than 100 different projects, as well as in routine work in occupational health care services. More than 50 000 persons have responded to one or more of the questionnaires.

Reliability and validity of the results

The reliability and validity of the questionnaires has been investigated. Subjects have filled and refilled questionnaires

How to answer the questionnaire:

Please answer by putting a cross in the appropriate box — one cross for each question. You may be in doubt as to how to answer, but please do your best anyway. Please answer every question, even if you have never had trouble in any part of your body.

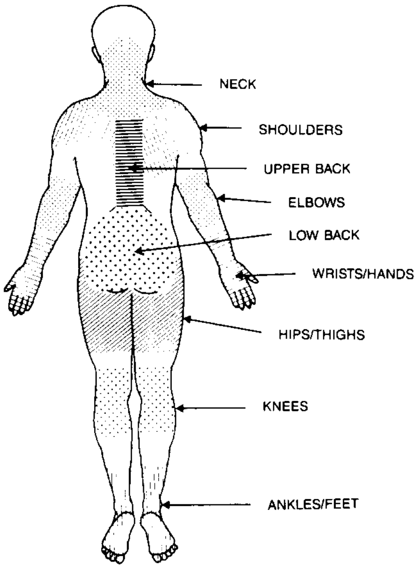

In this picture you can see the approximate position of the parts of the body referred to in the questionnaire. Limits are not sharply defined, and certain parts overlap. You should decide for yourself in which part you have or have had your trouble (if any).

Trouble with the locomotive organs

Have you at any time during the **last 12 months** had **trouble** (ache, pain, discomfort) in:	To be answered only by those who have had trouble: Have you at any time during the **last 12 months been prevented from doing your normal work** (at home or away from home) because of the trouble?	Have you had **trouble** at any time during the **last 7 days?**
**Neck**<br>1 No 2 Yes	1 No 2 Yes	1 No 2 Yes
**Shoulders**<br>1 No 2 Yes, in the right shoulder<br>3 Yes, in the left shoulder<br>4 Yes, in both shoulders	1 No 2 Yes	1 No 2 Yes
**Elbows**<br>1 No 2 Yes, in the right elbow<br>3 Yes, in the left elbow<br>4 Yes, in both elbows	1 No 2 Yes	1 No 2 Yes
**Wrists/hands**<br>1 No 2 Yes, in the right wrist/hand<br>3 Yes, in the left wrist/hand<br>4 Yes, in both wrists/hands	1 No 2 Yes	1 No 2 Yes
**Upper back**<br>1 No 2 Yes	1 No 2 Yes	1 No 2 Yes
**Low back (small of the back)**<br>1 No 2 Yes	1 No 2 Yes	1 No 2 Yes
**One or both hips/thighs**<br>1 No 2 Yes	1 No 2 Yes	1 No 2 Yes
**One or both knees**<br>1 No 2 Yes	1 No 2 Yes	1 No 2 Yes
**One or both ankles/feet**<br>1 No 2 Yes	1 No 2 Yes	1 No 2 Yes

and the subjects' responses to the questionnaires have been compared with their clinical history.

Reliability tests with the test-retest method of preliminary versions of the general questionnaire (one study on 29 safety engineers, one on 17 medical secretaries and one on 22 railway maintenance workers) showed that the number of non-identical answers varied from 0 to 23%. Validity tests against clinical history (one study on 19 medical secretaries and one on 20 railway maintenance workers) showed that the number of non-identical answers varied between 0 and 20%.

Questionnaire about low back trouble

The date of inquiry	____/____/____ year month day
Sex	1 Female 2 Male
What year were you born?	________
How many years and months have you been doing your present type of work?	____ years + ____ months
On average, how many hours a week do you work?	____ hours a week
How much do you weigh?	____ kg
How tall are you?	____ cm
Are you right-handed or left-handed?	1 right-handed<br>2 left-handed

NORDIC COUNCIL OF MINISTERS
PROJECT 170 21–330

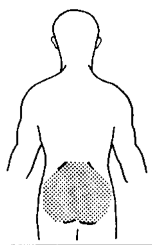

LOW BACK

How to answer the questionnaire: In this picture you can see the approximate position of the part of the body referred to in the questionnaire. By low back trouble is meant ache, pain or discomfort in the shaded area whether or not it extends from there to one or both legs (sciatica).

Please answer by putting a cross in the appropriate box — one cross for each question. You may be in doubt as to how to answer, but please do your best anyway.

1. Have you **ever** had low back trouble (ache, pain or discomfort)?
   1 No 2 Yes

If you answered **No** to Question 1, do not answer questions 2–8.

2. Have you **ever** been hospitalized because of low back trouble?
   1 No 2 Yes

3. Have you **ever** had to change **jobs or duties** because of low back trouble?
   1 No 2 Yes

4. What is the **total** length of time that you have had low back trouble during the **last 12 months?**
   1 0 days
   2 1–7 days
   3 8–30 days
   4 More than 30 days, but not every day
   5 Every day

If you answered **0 days** to question 4, do not answer the questions 5–8.

5. Has low back trouble caused you to **reduce** your activity during the **last 12 months?**
   a. Work activity (at home or away from home)?
   1 No 2 Yes
   b. Leisure activity?
   1 No 2 Yes

6. What is the **total length** of time that low back trouble **has prevented** you from doing your normal work (at home or away from home) during the **last 12 months?**
   1 0 days
   2 1–7 days
   3 8–30 days
   4 More than 30 days

7. Have you **been seen** by a doctor, physiotherapist, chiropractor or other such person because of low back trouble during the **last 12 months?**
   1 No 2 Yes

8. Have you had low back trouble at any time during the **last 7 days?**
   1 No 2 Yes

Fig. 2 Low back trouble questionnaire

The reliability of the neck-shoulder questionnaire was tested on 27 women in clerical work, who answered the questionnaire twice with a 3-week interval. The percentage of disagreeing responses varied from 0 to 15%, except for questions 4 and 13 (Fig. 3) where it was 30 and 22%, respectively. The validity was tested on 82 women in electronics manufacturing. The questionnaire responses were compared with those obtained when a physiotherapist filled out the questionnaire after a thorough interview about medical history. The percentage of disagreement between the subjects' own responses and the physiotherapist's estimates varied from 0 to 13%.

The reliability of a preliminary version of the low back questionnaire was tested on 25 nursing staff members who answered the questionnaire twice with a 15-day interval The percentage of disagreeing answers was on average 4·4, varying from 0 to 4%, except for one question where it was 25%. As a consequence, this question was reformulated in the final version.

The method of administration of the questionnaire has an effect on the response rates (Andersson *et al*, 1987).

The usage of the questionnaire

A critical question that arises is whether the questionnaires can provide useful information which can be used in decision-making in occupational health practice. Various studies have shown that response distributions are different for different occupational groups (Jonsson and Ydreborg, 1985) and that the differences are related to the estimated workload. In some studies the questionnaires have revealed a high prevalence of symptoms and disorders in certain anatomical regions which clearly correlate to the local physical demands (e g, Brulin *et al*, 1985).

The questionnaire has been structured for computer analysis. Routine analysis of various statistical epidemiological programmes can be applied. The dichotomy of the response alternatives may require special consideration (see, for example, Fleiss, 1973).

In the opinion of the project group the questionnaires provide useful and reliable information on musculoskeletal symptoms. This information either gives rise to further in-depth investigation or gives hints for decision-making on preventive measures.

Acknowledgement

The study was supported by the Nordic Council of Ministers, Oslo.

References

Anon
1960 (a) *British Medical Journal,* 2, 1665. Medical Research Council's Committee on the Aetiology of Chronic Bronchitis: Standardised questionnaires on respiratory symptoms.

Anon
1960 (b) *British Medical Journal,* Medical Research Council's Committee on the Aetiology of Chronic Bronchitis: Instructions for the use of the questionnaire on respiratory symptoms.

Questionnaire about neck and shoulder trouble

The date of inquiry — year month day

Sex — 1 Female 2 Male

What year were you born?

How many years and months have you been doing your present type of work? — years + months

On average, how many hours a week do you work? — hours a week

How much do you weigh? — kg

How tall are you? — cm

Are you right-handed or left-handed? — 1 right-handed 2 left-handed

NORDIC COUNCIL OF MINISTERS
PROJECT 170 21 · 330

Fig. 3 Neck and shoulder trouble questionnaire

Andersson, K., Karlehagen, S., and Jonsson, B.
1987 *Applied Ergonomics,* **18**, 3, 229–232. The importance of variations in questionnaire administration.

Armstrong, T.J., Foulke, J.A., Joseph, B.S., and Goldstein, S.A.
1982 *American Industrial Hygiene Association Journal,* **43**, 103–116. Investigation of cumulative trauma disorders in a poultry processing plant.

Brigham, F.R.
1975 *Applied Ergonomics,* **6**, 90–96. Some quantitative considerations in questionnaire design and analysis.

Brulin, C., Jonsson, B., and Karlehagen, S.
1985 Musculoskeletal trouble among Swedish railway workers (in Swedish). Arbete och Hälsa, (in press) Arbetarskyddsstyrelsen, Stockholm.

Corlett, E.N., and Bishop, R.P.
1978 *Applied Ergonomics,* **9**, 23–32. The ergonomics of spot welders.

Fleiss, J.L.
1973 Statistical methods for rates and proportions. John Wiley & Sons, New York.

Jonsson, B., and Ydreborg, B.
1985 Identification of ergonomic problems by means of questionnaires for musculoskeletal troubles. Proceedings of the 9th Congress of the International Ergonomics Association, Bournemouth, (Ed: I.D. Brown *et al*), 424–426.

Kilbom, A., Persson, J., and Jonsson, B.
1986 *International Journal of Industrial Ergonomics,* **1**, 37–47. Disorders of the cervicobrachial region among female workers in electronics industry.

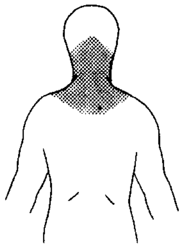

NECK

How to answer the questionnaire: By neck trouble is meant ache, pain or discomfort in the shaded area. Please concentrate on this area, ignoring any trouble you may have in adjacent parts of the body. There is a separate questionnaire for shoulder trouble.

Please answer by putting a cross in the appropriate box — one cross for each question. You may be in doubt as to how to answer, but please do your best anyway.

1. Have you **ever** had neck trouble (ache, pain or discomfort)?
   1 ☐ No 2 ☐ Yes

If you answered **No** to Question 1, do not answer the questions 2—8.

2. Have you **ever** hurt your neck in an **accident?**
   1 ☐ No 2 ☐ Yes
3. Have you **ever** had to change **jobs or duties** because of neck trouble?
   1 ☐ No 2 ☐ Yes
4. What is the **total** length of time that you have had neck trouble during the **last 12 months?**
   1 ☐ 0 days
   2 ☐ 1—7 days
   3 ☐ 8—30 days
   4 ☐ More than 30 days, but not every day
   5 ☐ Every day

If you answered **0 days** to Question 4, do not answer the questions 5—8.

5. Has neck trouble caused you to **reduce** your activity during the **last 12 months?**
   a. Work activity (at home or away from home)?
   1 ☐ No 2 ☐ Yes
   b. Leisure activity?
   1 ☐ No 2 ☐ Yes
6. What is the **total** length of time that neck trouble has prevented you from doing your normal work (at home or away from home) during the **last 12 months?**
   1 ☐ 0 days
   2 ☐ 1—7 days
   3 ☐ 8—30 days
   4 ☐ More than 30 days
7. Have you **been seen** by a doctor, physiotherapist, chiropractor or other such person because of neck trouble during the **last 12 months?**
   1 ☐ No 2 ☐ Yes
8. Have you had neck trouble at any time during the **last 7 days?**
   1 ☐ No 2 ☐ Yes

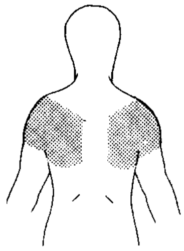

SHOULDER

How to answer the questionnaire: By shoulder trouble is meant ache, pain or discomfort in the shaded area. Please concentrate on this area, ignoring any trouble you may have in adjacent parts of the body. There is a separate questionnaire for neck trouble.

Please answer by putting a cross in the appropriate box — one cross for each question. You may be in doubt as to how to answer, but please do your best anyway.

9. Have you **ever** had shoulder trouble (ache, pain or discomfort)?
   1 No 2 Yes

If you answered **No** to Question 9, do not answer the questions 10—17.

10. Have you **ever** hurt your shoulder in an **accident?**
    1 No 2 Yes, my right shoulder
    3 Yes, my left shoulder
    4 Yes, both shoulders
11. Have you **ever** had to **change jobs or duties** because of shoulder trouble?
    1 No 2 Yes
12. Have you had shoulder trouble during the **last 12 months?**
    1 No 2 Yes, in my right shoulder
    3 Yes, in my left shoulder
    4 Yes, in both shoulders

If you answered **No** to Question 12, do not answer the questions 13—17.

13. What is the **total** length of time that you have had shoulder trouble during the **last 12 months?**
    1 ☐ 1—7 days
    2 ☐ 8—30 days
    3 ☐ More than 30 days, but not every day
    4 ☐ Every day
14. Has shoulder trouble caused you to **reduce** your activity during the **last 12 months?**
    a. Work activity (at home or away from home)?
    1 No 2 Yes
    b. Leisure activity?
    1 No 2 Yes
15. What is the **total** length of time that shoulder trouble has **prevented** you from doing your normal work (at home or away from home) during the **last 12 months?**
    1 0 days
    2 1—7 days
    3 8—30 days
    4 More than 30 days
16. Have you **been seen** by a doctor, physiotherapist, chiropractor or other such person because of shoulder trouble during the **last 12 months?**
    1 No 2 Yes
17. Have you had shoulder trouble at any time during the **last 7 days?**
    1 ☐ No 2 ☐ Yes, in my right shoulder
    3 ☐ Yes, in my left shoulder
    4 ☐ Yes, in both shoulders

Maeda, K., Hirayama, H., Chang, C-P., and Takamatsu, M.
1979 *Japanese Journal of Industrial Health,* 21, 398–407. Studies on the progress of occupational cervicobrachial disorder by analysing the subjective symptoms of workmen in assembly lines of a cigarette factory.

Oxenburgh, M.S., Rowe, S.A., and Douglas, D.B.
1985 *Journal of Occupational Health and Safety,* 2, 106–112. Repetition strain injury in keyboard operators – Australia and New Zealand.

Pope, M.H., Frymoyer, J., and Andersson, G.B.J.
1984 Occupational low back pain, Prager Press, Philadelphia.

Rose, G.A., and Blackburn, H.
1968 Cardiovascular survey methods, WHO, Geneva, 188.

Sinclair, M.A.
1975 *Applied Ergonomics,* **6**, 73–80. Questionnaire design.

Troup, J.D.G., and Edwards, F.C.
1985 Manual Handling. A review paper. Health and Safety Executive.

Van Wely, P.
1970 *Applied Ergonomics,* **1**, 258–261. Design and disease.

Waris, P.
1979 *Scandinavian Journal of Work, Environment & Health,* 5, Supplement 3, 3–14. Occupational cervicobrachial syndromes: A review.
