# Supplementary material for: Back pain in elite sports: A cross-sectional study on 1114 athletes
Source: PLoS One. 2017 Jun 29;12(6):e0180130. doi: 10.1371/journal.pone.0180130 (PMC5491135; doi:10.1371/journal.pone.0180130)
Supplement: S3 File — (PDF) [file pone.0180130.s003.pdf]

Fragebogen
über Beschwerden am Bewegungsapparat
(Nordischer Fragebogen)

Untersuchungsdatum		
Tag	Monat	Jahr

**Identnummer (ID)**<br>**(Bitte vom untersuchenden Arzt erfragen)**

Tätigkeitscode<br>(wird nachgetragen, bitte nicht ausfüllen)<br>(01 - 99)	

Hinweis zum Ausfüllen des Fragebogens:

Bitte lesen Sie die Fragen aufmerksam durch und beantworten Sie jede Frage nach Ihrer Einschätzung durch Einsetzen der Daten oder durch Ankreuzen der entsprechenden Antwortfelder. In Zweifelsfällen überlegen Sie bitte erneut und geben dann die am meisten zutreffende Antwort.

1. Bitte geben Sie Ihr Geschlecht an!

☐ 1 weiblich ☐ 2 männlich

2. Welche Nationalität haben Sie?

☐ 0 Deutsch ☐ 1 andere

....

4. Wie alt sind Sie?

Lebensalt .... Jahre

5. Wie wird Ihre jetzige Tätigkeit bezeichnet?

....

5. Wie lange haben Sie diese Tätigkeit bei Ihrem <u>derzeitigen</u> Arbeitgeber ausgeübt?

ca. .... Jahre .... Monate

6. Wie lange haben Sie diese Tätigkeit <u>insgesamt</u>, also auch bei anderen Arbeitgebern ausgeübt?

ca. .... Jahre .... Monate

7. Wieviele Stunden arbeiten Sie im Durchschnitt in der Woche (Regelarbeitszeit + Überstunden)?

.... Stunden

8. Welche anderen Tätigkeiten haben Sie in Ihrem Leben bisher ausgeübt (Lehre, Studium, Wehrdienst, Berufstätigkeiten usw.)? Wie lange dauerten diese? Falls der vorgesehene Platz in der Tabelle nicht ausreicht, verwenden Sie bitte die Rückseite dieses Bogens.

Zeitraum von - bis	Bezeichnung der Tätigkeit

Ihre Bemerkungen:

Fragebogen zu Beschwerden am Stütz- und Bewegungsapparat

1. Gab es bisher einen Arbeitsplatzwechsel aus gesundheitlichen Gründen ?

☐ 0 Nein ☐ 1 Ja

2. Wieviel wiegen Sie ?

Ihr Körpergewicht: ca. .... kg

3. Wie groß sind Sie?

Ihre Körpergröße : ca. .... cm:

4. Sind Sie Rechts- oder Linkshänder?

☐ 1 Linkshänder ☐ 2 Rechtshänder

5. Treiben Sie regelmäßig Sport? Wenn ja, welchen?

☐ 0 Nein ☐ 1 Ja

Sportart ....

ca. .... Stunden pro Woche

6. Rauchen Sie?

☐ 0 Nein ☐ 1 Ja

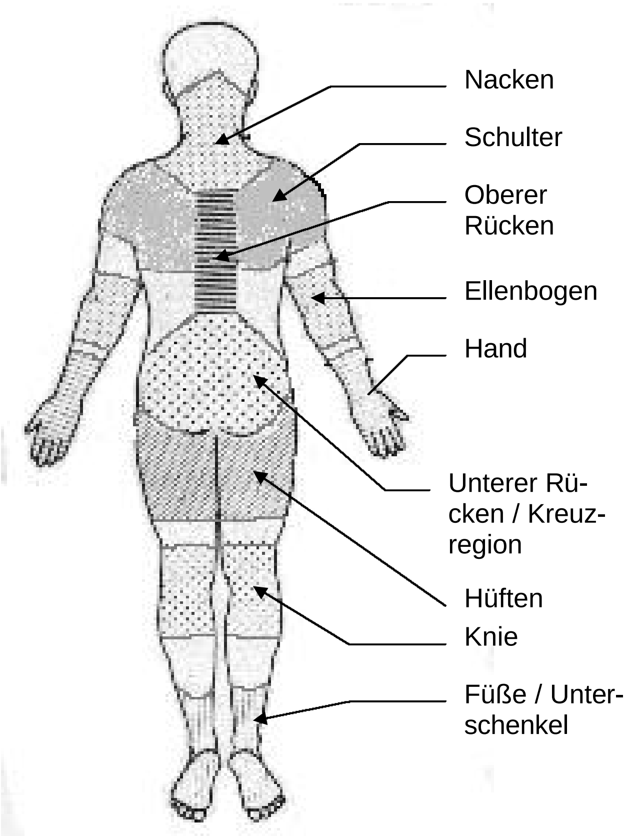

In den nächsten Fragen sollen Sie angeben, ob Sie in einem bestimmten Körperbereich schon einmal Schmerzen oder Beschwerden hatten. Die Körperregionen, die auf den nächsten Seiten abgefragt werden, wurden in der links abgebildeten kleinen Figur eingezeichnet.

Die Grenzen zwischen den Körperregionen können auch ineinander übergehen. Sie entscheiden bitte selbst, welche Körperbereiche betroffen sind.

Unter Schmerzen sind sowohl punktförmige und eng begrenzte Schmerzen bis hin zu nicht genau lokalisierbaren und nicht genau beschreibbaren Schmerzempfindungen in den angegebenen Körperregionen zu verstehen.

Bitte berücksichtigen Sie auch Schmerzen, wenn Sie in einen anderen Körperbereich (z.B. ein Ischias-Schmerz) ausstrahlen! In Zweifelsfällen versuchen Sie bitte die am meisten zutreffende Antwort zu geben!

Allgemeine Angaben zu Beschwerden im Muskel-Skelett-System

Hatten Sie während der letzten 12 Monate zu irgend einer Zeit Beschwerden oder Schmerzen in folgenden Körperregionen?	**Diese beiden Spalten sind nur zu beantworten, wenn die Fragen in der 1. Spalte (links) mit „Ja" beantwortet wurden.**	
	Waren sie wegen der Beschwerden in den letzten 12 Monaten irgendwann nicht in der Lage, ihre normale Arbeit zu tun (beruflich, zu Hause oder Freizeitbeschäftigungen)?	Hatten Sie während der letzten 7 Tage irgendwann Beschwerden?
**1. Nackenregion**<br>☐ 0 Nein ☐ 1 Ja	☐ 0 Nein ☐ 1 Ja	☐ 0 Nein ☐ 1 Ja
**2. Schulterregion**<br>☐ 0 Nein ☐ 1 Ja, rechts<br>☐ 2 Ja, links<br>☐ 3 Ja, beidseits	☐ 0 Nein ☐ 1 Ja	☐ 0 Nein ☐ 1 Ja
**3. Ellenbogenregion**<br>☐ 0 Nein ☐ 1 Ja, rechts<br>☐ 2 Ja, links<br>☐ 3 Ja, beidseits	☐ 0 Nein ☐ 1 Ja	☐ 0 Nein ☐ 1 Ja
**4. Handgelenke / Hände**<br>☐ 0 Nein ☐ 1 Ja, rechts<br>☐ 2 Ja, links<br>☐ 3 Ja, beidseits	☐ 0 Nein ☐ 1 Ja	☐ 0 Nein ☐ 1 Ja
**5. Oberer Rücken / Brustwirbelsäule**<br>☐ 0 Nein ☐ 1 Ja	☐ 0 Nein ☐ 1 Ja	☐ 0 Nein ☐ 1 Ja
**6. Unterer Rücken (Kreuz)**<br>☐ 0 Nein ☐ 1 Ja	☐ 0 Nein ☐ 1 Ja	☐ 0 Nein ☐ 1 Ja
**7. Ein oder beide Hüften / Oberschenkel**<br>☐ 0 Nein ☐ 1 Ja	☐ 0 Nein ☐ 1 Ja	☐ 0 Nein ☐ 1 Ja
**8. Ein oder beide Knie**<br>☐ 0 Nein ☐ 1 Ja	☐ 0 Nein ☐ 1 Ja	☐ 0 Nein ☐ 1 Ja
**9. Ein oder beide Knöchel / Füße**<br>☐ 0 Nein ☐ 1 Ja	☐ 0 Nein ☐ 1 Ja	☐ 0 Nein ☐ 1 Ja

<u>Fragen zu Beschwerden in der Nackenregion / Halswirbelsäule</u>

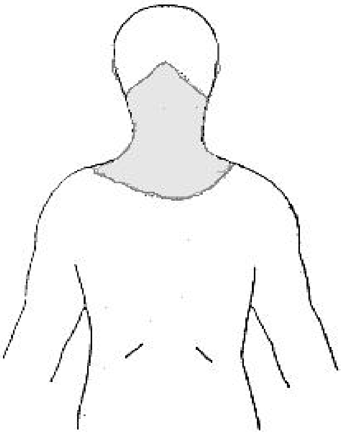

Unter **Nackenbeschwerden** werden Stechen, Schmerzen und Mißempfindungen in dem schraffierten Gebiet zusammengefaßt.

Bitte konzentrieren Sie sich ausschließlich auf diesen Bereich, berücksichtigen Sie nicht Beschwerden, die in benachbarten Körperteilen auftreten. Solche Beschwerden, z.B. im Schultergebiet, werden gesondert erfragt.

Bitte beantworten Sie die Fragen durch Einsetzen von Kreuzen in die Antwortfelder - <u>ein</u> Kreuz für jede Frage. In Zweifelsfällen versuchen Sie die am meisten zutreffende Antwort zu geben.

1. Hatten Sie irgendwann in Ihrem Leben Beschwerden im Nacken bzw. im Bereich der Halswirbelsäule (schraffierter Bereich)?

☐ 0 Nein
Falls Sie keine Beschwerden hatten, fahren Sie bitte mit der Beantwortung auf der folgenden Seite bei Frage 1 fort.

☐ 1 Ja weiter

2. Waren Sie jemals wegen dieser Nackenbeschwerden im Krankenhaus?

☐ 0 Nein ☐ 1 Ja

3. Wurden Sie im Bereich des Nackens während eines Unfalles verletzt?

☐ 0 Nein ☐ 1 Ja

4. Mußten Sie aufgrund von Nackenbeschwerden irgendwann einmal Ihre Arbeitsstelle oder berufliche Tätigkeit wechseln?

☐ 0 Nein ☐ 1 Ja

5. Bitte geben Sie an, wie lange Sie in den letzten 12 Monaten insgesamt Nackenbeschwerden verspürt haben! Falls Sie mehrfach krank waren, addieren Sie bitte alle Zeitabschnitte.

Beschwerden im Nacken hatte ich in den letzten 12 Monaten...

☐ 0 niemals (bzw. 0 Tage).
☐ 1 an 1-7 Tagen.
☐ 2 an 8-30 Tagen.
☐ 3 an mehr als 30 Tage, jedoch nicht täglich.
☐ 4 jeden Tag.

Falls Sie im letzten Jahr <u>keine</u> Nackenschmerzen hatten, fahren Sie bitte bei Frage 1 auf der nächsten Seite fort.

Falls Sie jedoch im letzten Jahr Nackenschmerzen hatten, beantworten Sie bitte auch die folgenden Fragen! weiter

6. Haben die Nackenbeschwerden Sie veranlaßt, Ihre Aktivitäten während der letzten 12 Monate einzuschränken?

A. Arbeitsaktivitäten (Berufstätigkeit oder Hausarbeit)

☐ 0 Nein ☐ 1 Ja

B. Freizeitaktivitäten

☐ 0 Nein ☐ 1 Ja

7. Über welche Zeitspanne haben die Nackenbeschwerden Ihre normale Arbeit (beruflich oder Hausarbeit) während der letzten 12 Monate behindert?

☐ 0 So stark waren die Beschwerden nicht.
☐ 1 an 1-7 Tagen.
☐ 2 an 8-30 Tagen.
☐ 3 an mehr als 30 Tagen.

8. Haben Sie wegen Ihrer Nackenbeschwerden einen Arzt, einen Chiropraktiker oder Physiotherapeuten o.ä. aufgesucht?

☐ 0 Nein ☐ 1 Ja

9. Hatten Sie in der letzten Woche bzw. in den letzten 7 Tagen irgendwann Beschwerden in der Nackenregion?

☐ 0 Nein ☐ 1 Ja

Fragen zu Beschwerden im unteren Rücken (Kreuz / Lendenwirbelsäule)

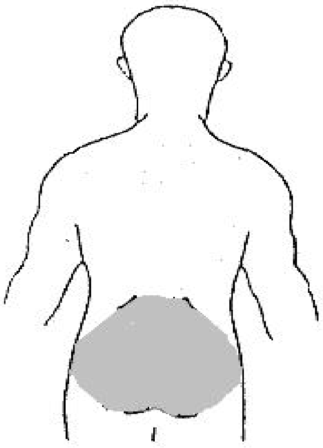

Die folgenden Fragen beziehen sich auf den schraffierten Körperteil. Bei Beschwerden im Kreuz sind z.B. gemeint: lokaler punktförmiger Schmerzen, nicht genau lokalisierbare Schmerzen oder nicht genau zu beschreibende Schmerzempfindungen in dem schraffierten Gebiet, unabhängig davon, ob der Schmerz in ein Bein oder beide Beine ausstrahlt (Ischias).

Bitte beantworten Sie die Fragen durch Einsetzen von Kreuzen in die Antwortfelder - ein Kreuz für jede Frage. In Zweifelsfällen versuchen Sie die am meisten zutreffende Antwort zu geben.

1. Hatten Sie irgendwann in Ihrem Leben Beschwerden im Kreuz bzw. im Bereich der Lendenwirbelsäule (schraffierter Bereich)?

☐ 0 Nein
Falls Sie keine Beschwerden hatten, fahren Sie bitte auf der folgenden Seite bei Frage 1 fort.

☐ 1 Ja weiter

2. Waren Sie jemals wegen Ihrer Rückenbeschwerden im Krankenhaus?

☐ 0 Nein ☐ 1 Ja

3. Wurden Sie im Bereich der Lendenwirbelsäule während eines Unfalles verletzt?

☐ 0 Nein ☐ 1 Ja

4. Mußten Sie aufgrund von Rückenbeschwerden irgendwann einmal Ihre Arbeitsstelle oder berufliche Tätigkeit wechseln?

☐ 0 Nein ☐ 1 Ja

5. Bitte geben Sie an, wie lange Sie in den letzten 12 Monaten insgesamt Rückenbeschwerden verspürt haben! Falls Sie mehrfach krank waren, addieren Sie bitte alle Zeitabschnitte!

Beschwerden im Rücken hatte ich in den letzten 12 Monaten...

☐ 0 niemals (bzw. 0 Tage).
☐ 1 an 1-7 Tagen.
☐ 2 an 8-30 Tagen.
☐ 3 an mehr als 30 Tage, jedoch nicht täglich.
☐ 4 jeden Tag.

Falls Sie im letzten Jahr keine Rückenbeschwerden hatten, fahren Sie bitte bei Frage 1 auf der nächsten Seite fort.

Falls Sie jedoch im letzten Jahr Rückenbeschwerden hatten, beantworten Sie bitte auch die folgenden Fragen !

weiter

6. Haben die Rückenbeschwerden Sie veranlaßt, Ihre Aktivitäten während der letzten 12 Monate einzuschränken?

A. Arbeitsaktivitäten (Berufstätigkeit oder Hausarbeit)

☐ 0 Nein ☐ 1 Ja

B. Freizeitaktivitäten

☐ 0 Nein ☐ 1 Ja

7. Über welche Zeitspanne haben die Rückenbeschwerden Ihre normale Arbeit (beruflich oder Hausarbeit) während der letzten 12 Monate behindert?

☐ 0 So stark waren die Beschwerden nicht.
☐ 1 an 1-7 Tagen.
☐ 2 an 8-30 Tagen.
☐ 3 an mehr als 30 Tagen.

8. Haben Sie wegen Ihrer Rückenbeschwerden einen Arzt, einen Chiropraktiker oder Physiotherapeuten o.ä. aufgesucht?

☐ 0 Nein ☐ 1 Ja

9. Hatten Sie in der letzten Woche bzw. in den letzten 7 Tagen irgendwann Rückenbeschwerden?

☐ 0 Nein ☐ 1 Ja

Fragen zu Beschwerden in den Schultern

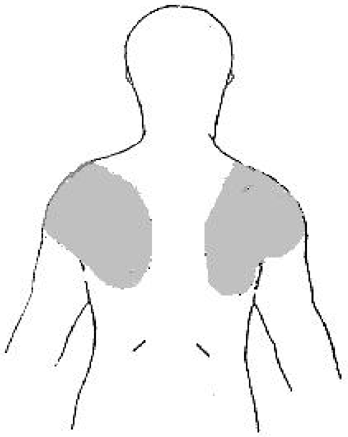

Unter **Schulterbeschwerden** werden Stechen, Schmerzen und Mißempfindungen in dem oben gekennzeichneten Bereich zusammengefaßt.

Bitte konzentrieren Sie sich hier ausschließlich auf diesen Bereich, berücksichtigen Sie nicht Beschwerden, die in benachbarten Bereichen (z.B. Nacken) auftreten.

Bitte beantworten Sie die Fragen durch Einsetzen von Kreuzen in die Antwortfelder - ein Kreuz für jede Frage. In Zweifelsfällen versuchen Sie die am meisten zutreffende Antwort zu geben.

1. Hatten Sie irgendwann in Ihrem Leben Beschwerden in den Schultern (schraffierter Bereich) ?

☐ $_0$ Nein
Falls Sie <u>keine</u> Beschwerden hatten, entfällt die Beantwortung der folgenden Fragen.

☐$_1$ Ja, links ☐$_2$ Ja, rechts ☐$_3$ Ja, beidseitig

weiter

2. Waren Sie jemals wegen Ihrer Schulterbeschwerden im Krankenhaus?

☐ $_0$ Nein ☐ $_1$ Ja

3. Wurden Sie im Bereich der Schultern während eines Unfalles verletzt?

☐$_0$ Nein
☐ $_1$ Ja, links
☐ $_2$ Ja, rechts
☐ $_3$ Ja, beidseitig

4. Mußten Sie aufgrund von Schulterbeschwerden irgendwann einmal Ihre Arbeitsstelle oder berufliche Tätigkeit wechseln?

☐ $_0$ Nein ☐ $_1$ Ja

5. Bitte geben Sie an, wie lange Sie in den letzten 12 Monaten Schulterbeschwerden verspürt haben! Falls Sie mehrfach krank waren, addieren Sie bitte alle Zeitabschnitte!

Beschwerden in den Schultern hatte ich in den letzten 12 Monaten...

☐ $_0$ niemals (bzw. 0 Tage).
☐ $_1$ an 1-7 Tagen.
☐ $_2$ an 8-30 Tagen.
☐ $_3$ an mehr als 30 Tage, jedoch nicht täglich.
☐ $_4$ jeden Tag.

Falls Sie im letzten Jahr <u>keine</u> Schulterbeschwerden hatten, entfällt die Beantwortung der folgenden Fragen.

Falls Sie jedoch im letzten Jahr Schulterbeschwerden hatten, beantworten Sie bitte auch die folgenden Fragen!

weiter

6. Haben die Schulterbeschwerden Sie veranlaßt, Ihre Aktivitäten während der letzten 12 Monate einzuschränken?

A. Arbeitsaktivitäten (Berufstätigkeit oder Hausarbeit)

☐ $_0$ Nein ☐ $_1$ Ja

B. Freizeitaktivitäten

☐ $_0$ Nein ☐ $_1$ Ja

7. Über welche Zeitspanne haben die Schulterbeschwerden Ihre normale Arbeit (beruflich oder Hausarbeit) während der letzten 12 Monate behindert?

☐ $_0$ So stark waren die Beschwerden nicht.
☐ $_1$ an 1-7 Tagen.
☐ $_2$ an 8-30 Tagen.
☐ $_3$ an mehr als 30 Tagen.

8. Haben Sie wegen Ihrer Schulterbeschwerden einen Arzt, einen Chiropraktiker oder Physiotherapeuten o.ä. aufgesucht?

☐ $_0$ Nein ☐ $_1$ Ja

9. Hatten Sie in der letzten Woche bzw. in den letzten 7 Tagen irgendwann Schulterbeschwerden?

☐ $_0$ Nein ☐ $_1$ Ja

Vielen Dank für Ihre Mitarbeit.
