# Supplementary material for: Back pain in elite sports: A cross-sectional study on 1114 athletes
Source: PLoS One. 2017 Jun 29;12(6):e0180130. doi: 10.1371/journal.pone.0180130 (PMC5491135; doi:10.1371/journal.pone.0180130)
Supplement: S5 File — (DOCX) [file pone.0180130.s005.docx]

1. Welcher Kategorie lässt sich Ihre Hauptsportart zuordnen?

What kind of sport are you doing?

1. Seit wie vielen Jahren üben Sie Ihre Hauptsportart/ Ihre Hauptsportarten aus? (in Jahren)

How many years have you been practicing your main sport?

1. Was ist Ihr derzeitiges Wettkampfniveau (z.B. A-, B-, C-Kader, 1. Bundesliga etc.)?

What is your current level of competition?

1. Wie häufig und wie lange trainieren Sie etwa in einer Trainingswoche?

How often and how long do you train during the week?
